# Supplementary material for: Genome-Wide Analysis Reveals Human-Mediated Introgression from Western Pigs to Indigenous Chinese Breeds
Source: Genes (Basel). 2020 Mar 4;11(3):275. doi: 10.3390/genes11030275 (PMC7140852; doi:10.3390/genes11030275)
Supplement: Supplementary file 1 [file genes-11-00275-s001.zip › genes-710665 supplementary.docx]

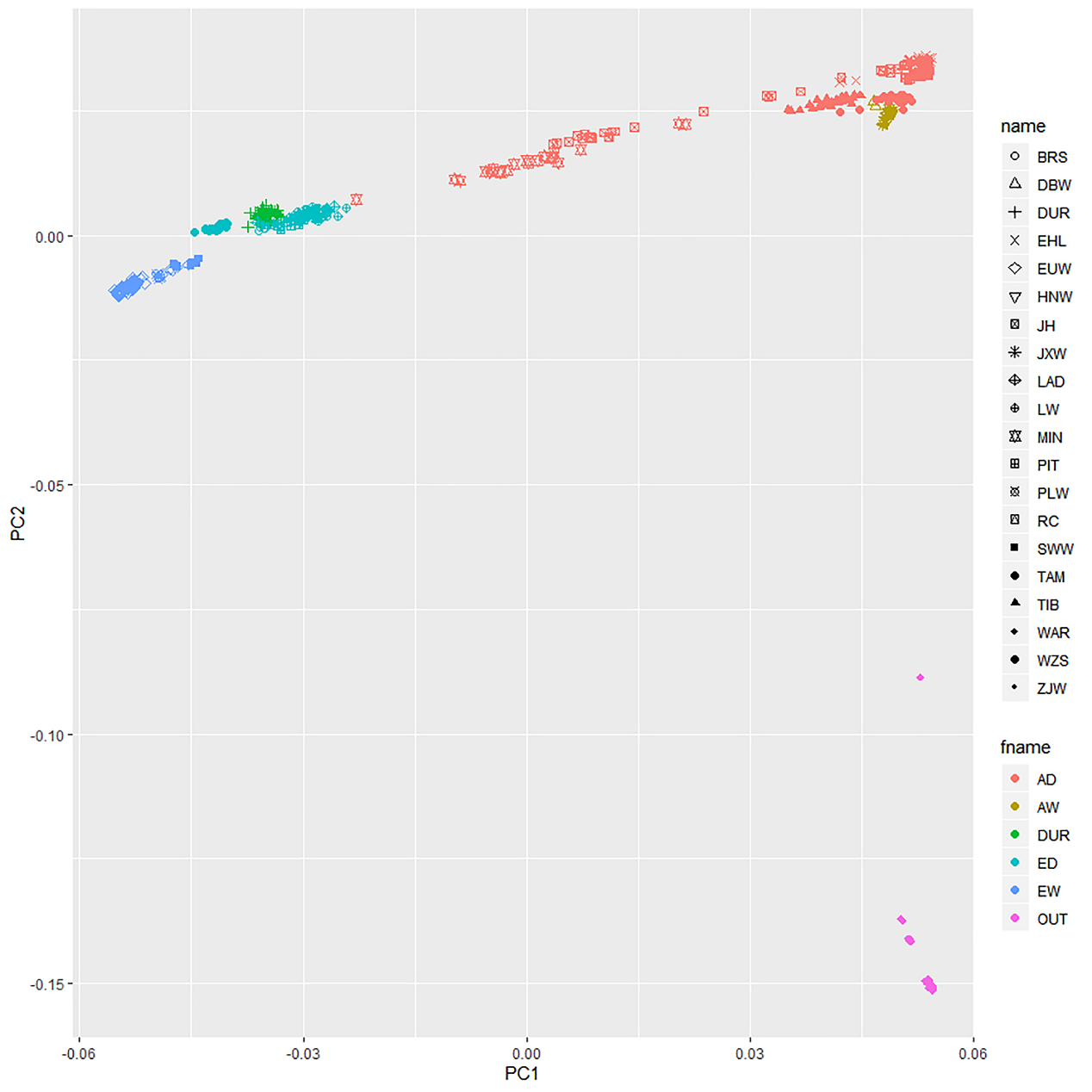


Figure S1. PCA result including outgroup in PC1 and PC2


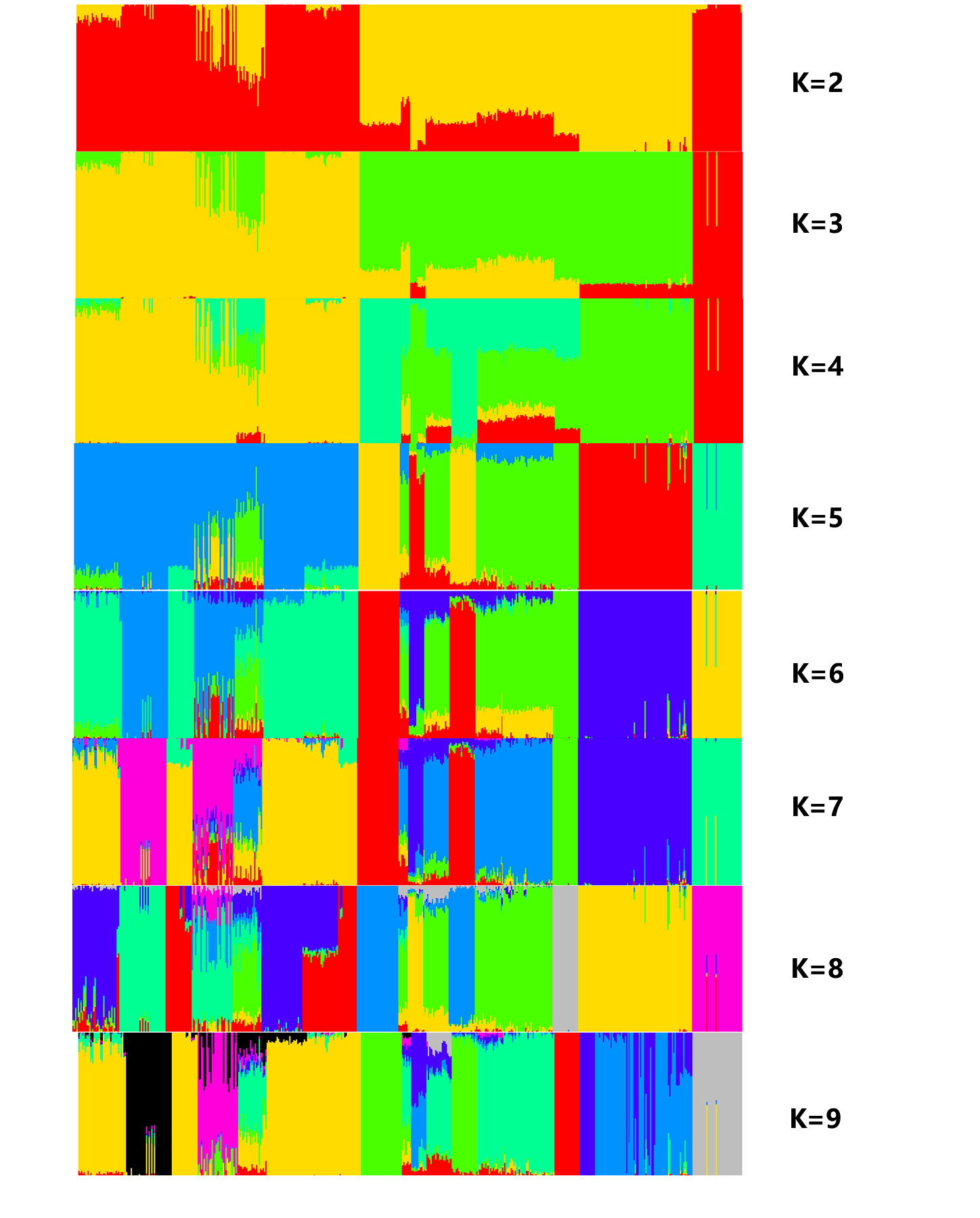


Figure S2. Genetic structure result including outgroup (located in far right of the plots) from 2 to 9


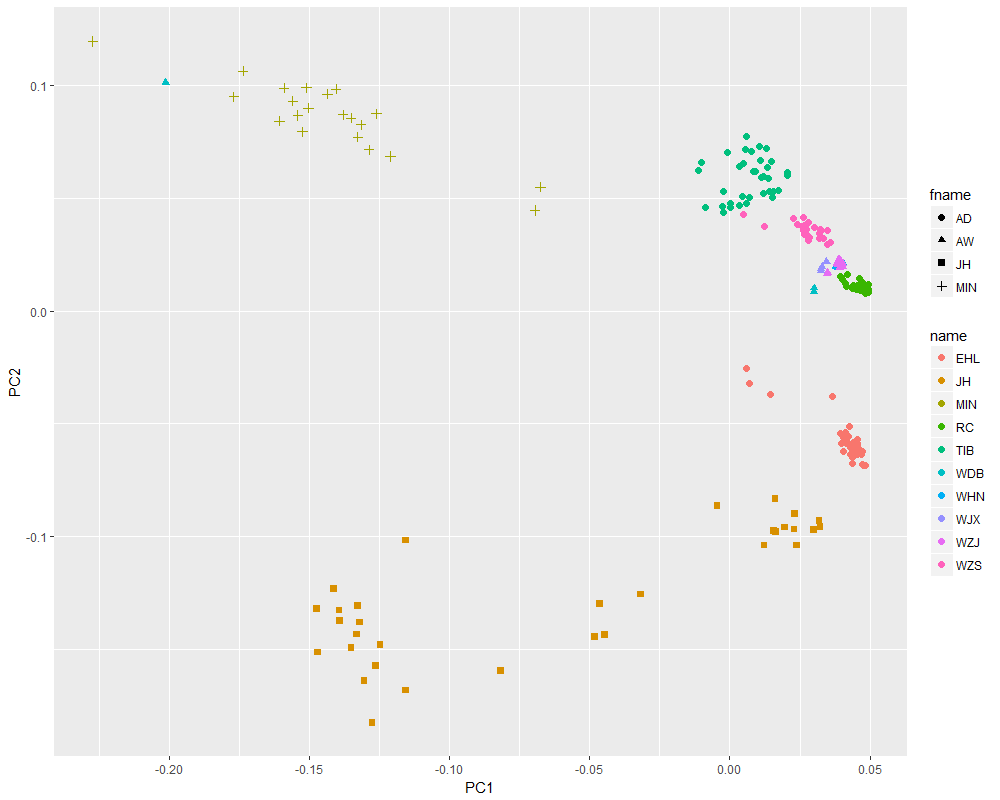


Figure S3. PCA result of Asian pigs.
